# CE-MS/MS and CE-timsTOF to separate and characterize intramolecular disulfide bridges of monoclonal antibody subunits and their application for the assessment of subunit reduction protocols

**DOI:** 10.1007/s00216-024-05161-8

**Published:** 2024-02-01

**Authors:** Jasmin Schairer, Jennifer Römer, Dietmar Lang, Christian Neusüß

**Affiliations:** 1 Faculty of Chemistry, Aalen University, Aalen, Germany; 2 Faculty of Science, University of Tübingen, Tübingen, Germany; 3 Rentschler Biopharma SE, Laupheim, Germany

**Keywords:** mAb subunits, Disulfides, Reduction, Capillary electrophoresis, Middle down, Ion mobility

## Abstract

Characterization at the subunit level enables detailed mass spectrometric characterization of posttranslational modifications (PTMs) of monoclonal antibodies (mAbs). The implemented reduction often leaves the intramolecular disulfide bridges intact. Here, we present a capillary electrophoretic (CE) method based on a neutral-coated capillary for the separation of immunoglobulin G-degrading enzyme of *Streptococcus pyogenes* (IdeS) digested and reduced mAb subunits followed by mass spectrometry (MS), MS/MS identification, and trapped ion mobility mass spectrometry (timsTOF). Our CE approach enables the separation of (i) different subunit moieties, (ii) various reduction states, and (iii) positional isomers of these partly reduced subunit moieties. The location of the remaining disulfide bridges can be determined by middle-down electron transfer higher energy collisional dissociation (EThcD) experiments. All these CE-separated variants show differences in ion mobility in the timsTOF measurements. Applying the presented CE-MS/MS method, reduction parameters such as the use of chaotropic salts were studied. For the investigated antibodies, urea improved the subunit reduction significantly, whereas guanidine hydrochloride (GuHCl) leads to multiple signals of the same subunit in the CE separation. The presented CE-MS method is a powerful tool for the disulfide-variant characterization of mAbs on the subunit level. It enables understanding disulfide bridge reduction processes in antibodies and potentially other proteins.

**Supplementary Information:**

The online version contains supplementary material available at 10.1007/s00216-024-05161-8.

## Introduction

Monoclonal antibodies (mAbs) are important biotherapeutics applied for the treatment of many human diseases, with anticancer therapy being one of the main fields [1]. The characterization of mAbs is important because changes in the structure or posttranslational modifications (PTMs) can happen due to production or storage [2]. These critical quality attributes (CQA), such as the glycosylation pattern, deamidation, disulfide bridges, C-terminal lysin clipping, pyroglutamate, or oxidation, can change the mAbs stability, efficacy, structure, and functionality [3–8]. Proper disulfide bridge formation is vital for the antibodies’ structure [9], stability [9, 10], and functionality [8].

Depending on the desired information, mAbs can be analyzed at an intact, reduced, subunit, or peptide level [11]. On the peptide level, the CQAs can be analyzed in great detail, and small changes can be detected [12, 13] even though sample preparation is quite elaborate and artifacts might occur. The approach with the least sample preparation is the analysis on an intact level. However, small changes are difficult to characterize by mass spectrometry (MS) [11, 14]. As a compromise between the intact and peptide level, mAbs can be reduced or enzymatically cut to their subunit moieties. The subunit term is not specified, so several approaches exist to obtain subunits. If the mAb is reduced, heavy chain (HC, 50 kDa) and light chain (LC, 25 kDa) subunits can be analyzed [15, 16]. Enzymes that cleaves the mAb underneath the hinge region [17] produce the C-terminal half of heavy chain (Fc/2; 25 kDa) and the antigen-binding fragment (F(ab)_2_; 100 kDa) subunits [16, 18, 19]. If these moieties are reduced, the N-terminal half of the heavy chain (Fd), LC, and Fc/2 are received, each with a molecular weight of ~ 25 kDa [11, 16, 20]. To obtain the 25 kDa subunits, different approaches can be done. The initial digestion using the immunoglobulin G-degrading enzyme of *Streptococcus pyogenes* (IdeS) is well established (standard procedure uses 1 U enzyme/1 µg antibody followed by incubation for 30 min at 37 °C). However, the following reduction to obtain the 25 kDa subunits can be performed quite differently. Dithiothreitol (DTT) [16, 20–29] or tris(2-carboxyethyl)phosphine (TCEP) [11, 20, 23, 25, 30–34] are used as reduction chemicals, and the reduction is then carried out for 5–60 min at room temperature (RT) [11, 25, 30–33], 37 °C [21, 23, 26, 28, 29], 45 °C [27], 55–60 °C [16, 20, 22, 25, 34], or 70 °C [24]. The reduction can be performed in guanidine hydrochloride (GuHCl) [16, 20, 22, 23, 25, 31, 33, 34], urea [24, 32], ammonium formate [28], or without chaotropic salt [11, 21, 25–27, 29, 30]. The reduction can be followed by further steps like acidification using trifluoroacetic acid [31, 33], formic acid (FA) [32], or hydrochloric acid (HCl) [29] or rebuffering and desalting [11, 20, 23, 34]. It is also known that removing the reducing agent after reduction leads to scrambled disulfide bridge detection [30].

The main advantage of all these sample preparation steps is the ability to make the 25 kDa subunits accessible for middle-down MS/MS fragmentation experiments [25, 30, 31]. In contrast, fragmentation of the intact antibody requires dedicated equipment and delivers moderate fragmentation coverages [35, 36]. Various PTMs, like changes in the general glycosylation pattern, can be analyzed at the subunit level, as well as deamidation, C-terminal lysine clipping, pyroglutamate formation, disulfide bridges, and oxidation [5, 37]. Still, small mass differences require separated proteoforms. This especially applies to disulfide bridge analysis on the subunit level [20, 27].

For the separation of mAb proteoforms on the subunit level, different high-performance liquid chromatography (HPLC) approaches like hydrophilic interaction liquid chromatography (HILIC) or reversed-phase chromatography (RP) are used. In HILIC approaches, the works focus on glycosylation analysis due to the changes in hydrophilicity of the Fc/2 subunit depending on the sugar attached [27, 38, 39]. RP approaches focus on general mass analysis of subunits [27, 32, 34], glycosylation analysis [28, 31, 32, 34], and methionine oxidation analysis [22, 29]. The separation and analysis of charge variants like lysin clipping or sialylation is mostly done using 2D approaches. In the first dimension, charge variants are separated using ion exchange chromatography or HILIC, followed by an RP separation for desalting or reduction in the second dimension [21, 40–42]. Nevertheless, 2D setups are complex and need a high instrumental effort. Since charge variants change an antibody’s size and charge, they are prone to capillary electrophoresis (CE) separation. Minimal size and charge shifts can be analyzed [19, 30]. Disulfides that change the subunit size without changing its charge were separated by CE on a cationic capillary coating followed by MS characterization [30]. However, the location of the disulfide remained unknown.

Compared to CE, ion mobility mass spectrometers (IM-MS) also separate molecules based on their size-to-charge but in the gas phase [43]. IM-MS is, therefore, highly interesting to analyze protein folding and conformation [44]. It was already shown that LC and HC could be separated in a field asymmetric waveform ion mobility spectrometry (FAIMS) device without a chromatographic pre-separation [45]. Conformational variants of antibodies were analyzed using IM-MS [46, 47], as well as peptides with different disulfide bridge conformations [48]. To the best of our knowledge, ion mobility was not used to study the 25 kD subunits of mAbs.

Here, we present CE-MS/MS and CE-trapped ion mobility mass spectrometry (timsTOF) MS methods to separate and analyze the intramolecular disulfides on the mAbs subunit level using a neutral-coated capillary. Coupling the CE to the MS was straightforward using the nanoCEasy interface [49]. For complete sample reduction, different approaches were tested, as well as the applicability of the reduction towards other immunoglobulin G1 (IgG1) mAbs.

## Materials and methods

### Chemicals and materials

Trastuzumab (N1050H05; 21 mg/mL) was purchased from Evidentic GmbH (Berlin, Germany); mAb1 (21.7 mg/mL), mAb2 (18.5 mg/mL), and mAb3 (18.3 mg/mL) were kindly provided by Rentschler Biopharma SE (Laupheim, Germany). USP mAb003 (Cat. No. 1445595, LOT: F12980, 10 mg/mL), 3-(N-morpholino) propanesulfonic acid (MOPS)-buffer, 1,4-dithiothreitol (DTT), and hydrochloric acid (37%) were purchased from Sigma (Steinheim, Germany). IdeS protease was purchased from GENOVIS (FabRICATOR, 5000 units, Lund, Sweden). Urea (Ultrapure) and guanidine hydrochloride (99.5%) were purchased from Thermo Fisher Scientific (Dreieich, Germany). Isopropanol (IPA, LC–MS grade) and formic acid (FA, ≥ 98%) were purchased from Carl Roth GmbH & Co. KG (Karlsruhe, Germany). Sodium hydroxide (NaOH) and hydrofluoric acid (40%(v/v), HF) were purchased from Merck (Darmstadt, Germany). Polyethylene oxide (PEO, Mw: 1.000.000) was purchased from Alfa Aesar (Kandel, Germany).

Ultrapure water (18 MΩ*cm at 25 °C, SG Ultra Clear UV from Siemens Water Technologies, USA) was used for all solutions.

### Sample preparation

All mAbs were digested following the FabRICATOR digestion protocol from GENOVIS. Five thousand units of FabRICATOR were reconstituted in water at a concentration of 67 U/µL. 9.5 µL of trastuzumab (21 mg/mL) were digested in 87.5 µL100 mM MOPS buffer at pH 7.2 using 3 µL of IdeS, resulting in a final mAb concentration of 2 mg/mL. The sample was incubated for 30 min at 37 °C and 500 rpm.

After digestion, 9 µL digest was reduced using 6 µL 0.5 M DTT (freshly prepared) and 15 µL of reduction medium (8 M urea/16 M urea/8 M GuHCl/water). The final solution contains 0.6 mg/mL of mAb, 100 mM DTT, and 4 M/8 M reduction medium. The reduction was incubated for 60 min at 37 °C and 500 rpm. The samples were then transferred to a glass inlet for CE vials and frozen at − 20 °C until measurement.

### CE analysis

The experiments were conducted on an Agilent 7100 CE instrument (Agilent Technologies GmbH, Waldbronn, Germany). Fused silica capillaries (separation capillary: 50 μm inner diameter (ID), 365 μm outer diameter (OD), and sheath liquid (SL) capillary: 100 μm ID, 240 μm OD) were purchased from Polymicro Technologies (Phoenix, AZ, USA).

An etched 60 cm length and 50 µm ID PEO-coated capillary was used for all experiments to avoid protein adsorption. For capillary etching, the polyimide of the separation capillary was removed at one end. The remaining glass was closed with hot glue and etched with HF (40% (v/v)) for 1 h to a thickness of < 150 µm [49]. The PEO coating procedure was adapted from Iki and Yeung [50]. For the PEO stock solution, 100 mg PEO was dissolved in 45 mL of water by heating the solution to 95 °C. 450 µL of the stock solution was then acidified using 50 µL 0.1 M HCl. The capillary was prepared with 1 M NaOH, water, and 1 M HCl for 5 min, respectively, followed by the PEO coating solution for 10 min, water, and background electrolyte (BGE) for 5 min, respectively. The solutions were flushed using the CE and an external pressure of 3 bar. The capillary coating was done each day to guarantee proper capillary coating.

The BGE consisted of 10% IPA in water containing 1 M FA. Sample injection was done hydrodynamically using 50 mbar pressure for 10 s. A 20 kV voltage was applied for the separation. The capillary was flushed for 3 min with BGE between the runs.

### nanoCEasy interface

For all CE-MS and CE-MS/MS experiments, the homebuilt nanoCEasy interface [49] was used. Emitters were obtained from BioMedical Instruments (Zoellnitz, Germany). The setup was controlled using a digital microscope (Dino-Lite, Almere, The Netherlands). The tip of the emitter had an opening of 30 µm and a tip length of 4 mm and was placed 3 mm in front of the MS orifice. In the separation mode, the separation capillary was placed 3 mm behind the emitter tip.

### MS and MS/MS and timsTOF analysis

To determine the m/z values for further fragmentation experiments of the subunits, MS experiments were performed using the Orbitrap Fusion Lumos Tribrid MS (Thermo Fisher Scientific, San Jose, CA, USA). IPA: Water (50:50) + 0.5% FA was used in all experiments as SL.

The MS was operated in positive ionization mode with a spray voltage of 2000 V, sweep gas of 3 Arb, and 300 °C ion transfer tube temperature. Data acquisition was done from 700 to 3000 m/z with an Orbitrap resolution of 120.000.

For MS/MS experiments, the Orbitrap was used at a resolution of 7500 in the MS1 scan. For MS2, the scan range was 150–2000 m/z with an Orbitrap resolution of 120,000. Electron transfer higher energy collisional dissociation (EThcD) was used to fragment the precursor ions. Electron transfer dissociation (ETD) reaction time was 12 ms (ETD reaction target of 6E5 in 200 ms), followed by 12% normalized higher energy collisional dissociation (HCD) collision energy.

A timsTOF SCP (Bruker Daltonics GmbH & Co.KG, Bremen, Germany) was used for IM-MS experiments. The instrument was operated in positive ionization mode with a capillary voltage of 1800 V, a dry gas flow of 3.0 L/min, and a dry temperature of 200 °C. The mobility (1/k0) was measured between 0.8 V*s/cm^2^ and 1.60 V*s/cm^2^ with a ramp time of 100 ms and an accumulation time of 20 ms.

### Data analysis

Orbitrap data were evaluated using Freestyle 1.5. To generate the extracted ion electropherograms (EIEs) of the subunits, the most intense charge states of each reduction state were summed up. The EIEs for the different reduction states were generated based on the most intense m/z of the charge envelope. Deconvolution of the Orbitrap MS data was done in Freestyle 1.5 using Xtract, setting the charge range from 5 to 50 and the minimum number of detected charges to 3. The MS/MS data was also deconvoluted using Xtract. The deconvoluted fragments were analyzed using ProSight Lite v1.4 (Northwestern University, Evanston, IL, USA) with a fragment tolerance of 10 ppm.

The timsTOF data were evaluated using DataAnalyst 5.3. For deconvolution, MaxEnt was used. Deconvolution was performed between 20,000 Da and 30,000 Da with a resolving power of 30,000 and normal resolution. Extracted ion mobilograms (EIMs) were generated for m/z values specifically.

The liquid phase charge of the subunits was determined using the Prot pi Protein Tool (https://www.protpi.ch/Calculator/ProteinTool) using the respective pH of the BGE and the sequence of the subunit.

## Results

### CE-MS

**Fig. 1:**
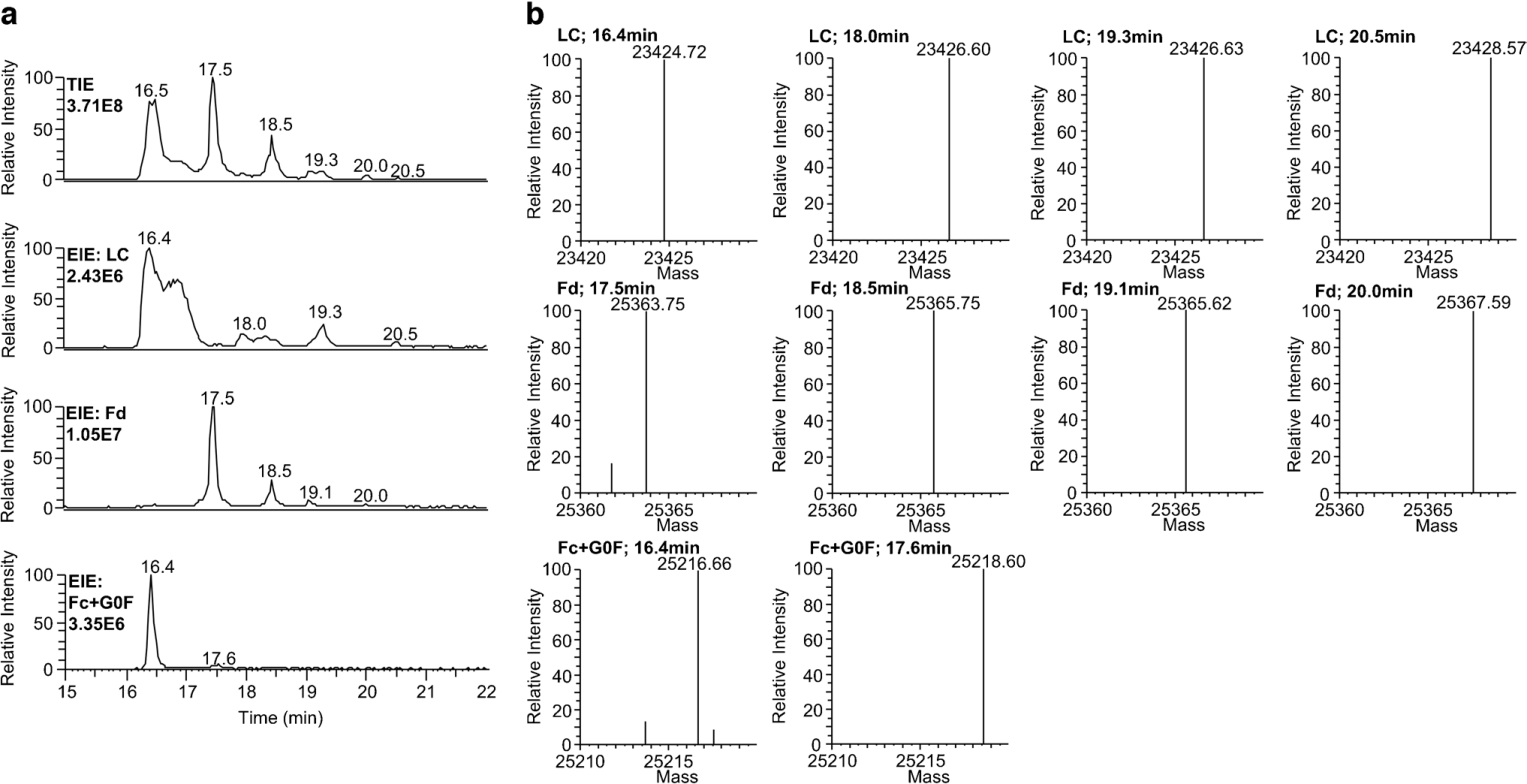
**a** Separation of trastuzumab subunits using the CE-MS system without fragmentation. EIEs are generated based on the sum of the two most intense m/z for each reduction state. Mass spectra of each subunit and reduction state are shown in Online Resource ESM 1. **b** Charge deconvoluted monoisotopic masses detected for each subunit and time point

Subunits can be generated using various reduction approaches. Initially, we digested trastuzumab with IdeS enzyme and further reduced the moieties without any chaotropic salt in water using DTT for 60 min at 37 °C. Three signals were expected based on the assumption that trastuzumab is digested and fully reduced to the three subunit moieties Fc/2 (G0F as the main glycoform is exemplarily used for the Fc/2 part of trastuzumab throughout the paper; 25,220 Da), Fd (25,367 Da), and LC (23,428 Da). However, several signals were observed in the total ion electropherogram (TIE), as shown in Fig. 1a. MS spectra can be found in Online Resource ESM 1.

The EIEs of the three moieties showed the separation of the subunits (Fig. 1a). LC and Fc/2 migrated simultaneously (16.4 min) but can be distinguished by their mass and m/z charge envelope, while Fd is well separated (17.5 min). For each subunit moiety, several signals appeared in their respective EIEs. For the LC masses of 23,424.72 Da, 23,426.60 Da, and 23,428.57 Da are obtained after deconvolution, with the latter being close to the theoretical fully reduced mass of 23,428.52 Da. The − 2 Da/ − 4 Da mass shift compared to the fully reduced mass can be expected to be due to an incomplete sample reduction. The same mass shifts were detected for Fd. For Fc/2, no fully reduced form was detected (see Fig. 1b). Another point to mention is that the mass shift of − 2 Da appeared two times in the EIE of the LC and Fd (18.0 min and 19.3 min for LC and 18.5 min and 19.1 min for Fd). To get a better understanding, especially of the − 2 Da shifted signals, MS/MS experiments were needed.

### CE-MS/MS

**Fig. 2:**
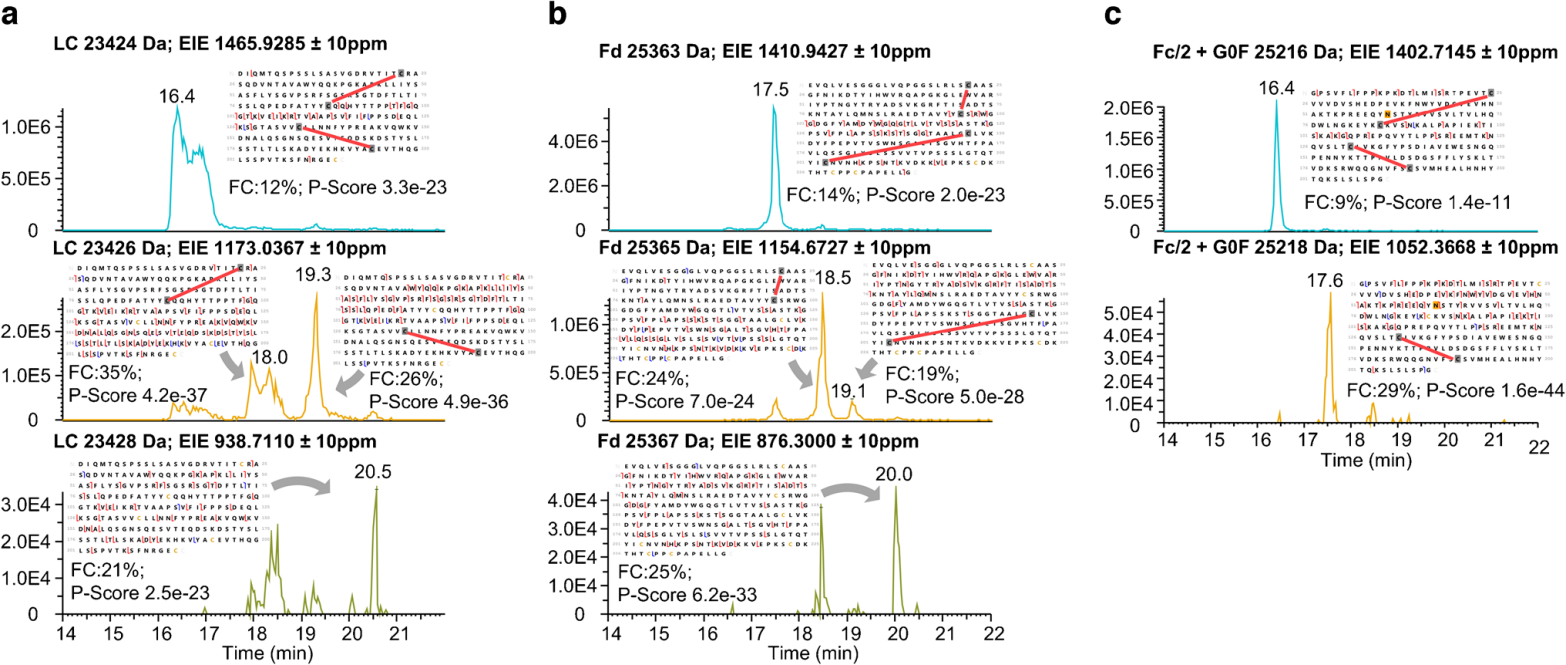
EIEs for the different reduction states for each subunit of trastuzumab **a** LC; **b** Fd, and **c** Fc/2. The EIE is generated based on the most intense m/z for each reduction state of each subunit. Based on the MS/MS experiment, fragmentation map, fragmentation coverage (FC), and P-score are given for each reduction stage. Red lines indicate closed disulfide bridges. m/z values chosen for EIE generation are marked in Online Resource ESM 1

MS/MS experiments were performed to confirm the ability of CE to characterize the different reduction states and to locate the oxidized and reduced disulfide bridges in the subunit moieties. Different fragmentation approaches were tested (collision-induced dissociation (CID), higher energy collisional dissociation (HCD), electron transfer dissociation (ETD), and electron transfer higher energy collisional dissociation (EThcD)), where EThcD yielded the best fragmentation coverage of the molecule (see ESM 2). Figure 2 shows the fragmentation results for each subunit and reduction state.

The first signal in the EIE of the LC (Fig. 2a) at 16.4 min showed a mass shift of − 4 Da, indicating no disulfide bridge reduction. The fragmentation result confirmed that the disulfide bridges in the variable light chain domain (V_L_; C23 to C88) and the constant light chain domain (C_L_; C134 to C194) remained intact. Fragments, one at the N terminus (*z* ion 212) and one at the C terminus (*c* ion 210), contributed to the fact that both disulfide bridges are closed. Fragments present between C88 and C134 indicated that no scrambling is present. The two peaks in the EIE, with a − 2 Da shift (18.0 min and 19.3 min), could be assigned to isomeric subunits with different disulfide bridge positions. For the signal at 18 min, the disulfide bridge in the C_L_ domain (C134 to C194) is open, showing a fragmentation coverage of 35%; for the signal at 19.3 min, the disulfide bridge in the V_L_ domain (C23 to C88) is open revealing a fragmentation coverage of 26%. This statement is confirmed by several fragments that can only appear if the respective disulfide bridge is open. No scrambled disulfide bridges were detected. However, low amounts of scrambled disulfide bridges would not be detected if they co-migrate with these two partly reduced LCs. The apparent double peaks in the EIE of LC are most likely related to ion suppression due to the co-migrating Fc/2 subunit at 16.4 min and the co-migrating Fd subunit at 18.5 min. The last signal in the EIE of the LC at 20.5 min indicated a full reduction based on mass. The MS/MS approach confirms this due to the detection of fragments that are only possible when both disulfides are open. The fragmentation coverage of 21% is low because the intensity of this reduction state is low using the sample preparation approach with no chaotropic salt. Higher fragmentation coverages with the same analysis were achieved in another sample preparation approach, which will be discussed later (see chapter sample preparation–reduction efficiency).

The same results described for the LC were achieved for Fd (Fig. 2b). Fd carries two disulfide bridges, one in the variable heavy chain domain (V_H)_ between C22 and C96 and the other in the constant heavy chain domain 1 (C_H_1) between C147 and C203. As shown for the LC, the non-reduced, the two partly reduced, and the fully reduced species were separated with CE and identified using MS/MS experiments.

For Fc/2 (Fig. 2c), no completely reduced subunit moiety was detected, and only one signal appeared for partly reduced species, which could be attributed to the Fc/2 subunit with a reduced disulfide bridge in the C_H_2-domain between C25 and C85 (C264 and C324 in HC).

All detected masses of the different reduction states are summarized in Table 1.

**Table 1:** All mAb subunits identified including positional isomers, their migration time, disulfide bond location, and theoretical and measured mass. Trastuzumab above the bold line was reduced in water, while trastuzumab and other mAbs below bold line were reduced in 4 M Urea. x*: no MS/MS data

mAb	subunit	Reduction state	Position of S–S bridge determined by MS/MS	Migration time [min]	Measured mass [monoisotopic]	Theoretical mass [monoisotopic]	Delta [ppm]
Trastuzumab	LC	2 S–S bridges	C23-C88 C134-C194	16.4	23,424.72	23,424.49	10
	LC	1 S–S bridge	C23-C88	18.0	23,426.60	23,426.51	4
	LC	1 S–S bridge	C134-C194	19.3	23,426.63	23,426.51	5
	LC	no S–S bridge	-	20.5	23,428.57	23,428.52	2
	Fd	2 S–S bridges	C22-C96 C147-C203	17.5	25,363.75	25,363.49	10
	Fd	1 S–S bridge	C22-C96	18.5	25,365.75	25,365.50	10
	Fd	1 S–S bridge	C147-C203	19.1	25,365.62	25,365.50	5
	Fd	no S–S bridge	-	20.0	25,367.59	25,367.52	3
	Fc/2	2 S–S bridges	C25-C85 C131-C189	16.4	25,216.66	25,216.43	9
	Fc/2	1 S–S bridge	C131-C189	17.6	25,218.60	25,218.44	6
Trastuzumab	LC	no S–S bridge	-	21.8	23,428.72	23,428.52	9
	Fd	no S–S bridge	-	23.3	25,367.73	25,367.52	8
	Fc/2	no S–S bridge	-	23.9	25,220.69	25,220.46	9
mAb1	LC	2 S–S bridges	x*	19.3	23,432.49	23,432.40	4
	LC	1 S–S bridge	C23-C88	21.1	23,434.49	23,434.42	3
	LC	no S–S bridge	-	23.0	23,436.53	23,436.43	4
	Fd	2 S–S bridges	x*	20.1	25,925.74	25,925.63	4
	Fd	1 S–S bridge	x*	21.2	25,927.70	25,927.64	2
	Fd	no S–S bridge	-	22.6	25,929.80	25,929.66	5
	Fc/2	2 S–S bridges	x*	18.8	25,216.47	25,216.43	2
	Fc/2	1 S–S bridge	C131-C189	20.0	25,218.59	25,218.45	6
	Fc/2	no S–S bridge	-	21.0	25,220.55	25,220.46	4
mAb2	LC	2 S–S bridges	C23-C93 C139-C199	20.6	23,887.84	23,887.77	3
	LC	1 S–S bridge	C23-C93	21.8	23,889.85	23,889.79	3
	LC	1 S–S bridge	C139-C199	22.3	23,889.91	23,889.79	5
	LC	no S–S bridge	-	23.5	23,891.89	23,891.80	4
	Fd	2 S–S bridges	x*	20.4	25,550.61	25,550.49	5
	Fd	1 S–S bridge	C148–C204	21.2	25,552.58	25,552.51	3
	Fd	1 S–S bridge	C22-C96	21.5	25,552.58	25,552.51	3
	Fd	no S–S bridge	-	22.0	25,554.61	25,554.52	4
	Fc/2	1 S–S bridge	C132-C190	20.5	25,257.61	25,257.51	4
	Fc/2	no S–S bridge	-	21.5	25,259.59	25,259.53	2
mAb3	LC	no S–S bridge	-	21.4	25,188.56	25,188.49	3
	Fd	no S–S bridge	-	22.9	25,631.63	25,631.53	4
	Fc/2	no S–S bridge	-	23.7	23,424.49	23,424.39	4
USP mAb003	LC	no S–S bridge	-	22.2	25,220.58	25,220.46	5
	Fd	no S–S bridge	-	24.0	25,250.64	25,250.47	7
	Fc/2	no S–S bridge	-	26.7	22,387.07	22,386.89	8

### CE-timsTOF MS

**Fig. 3:**
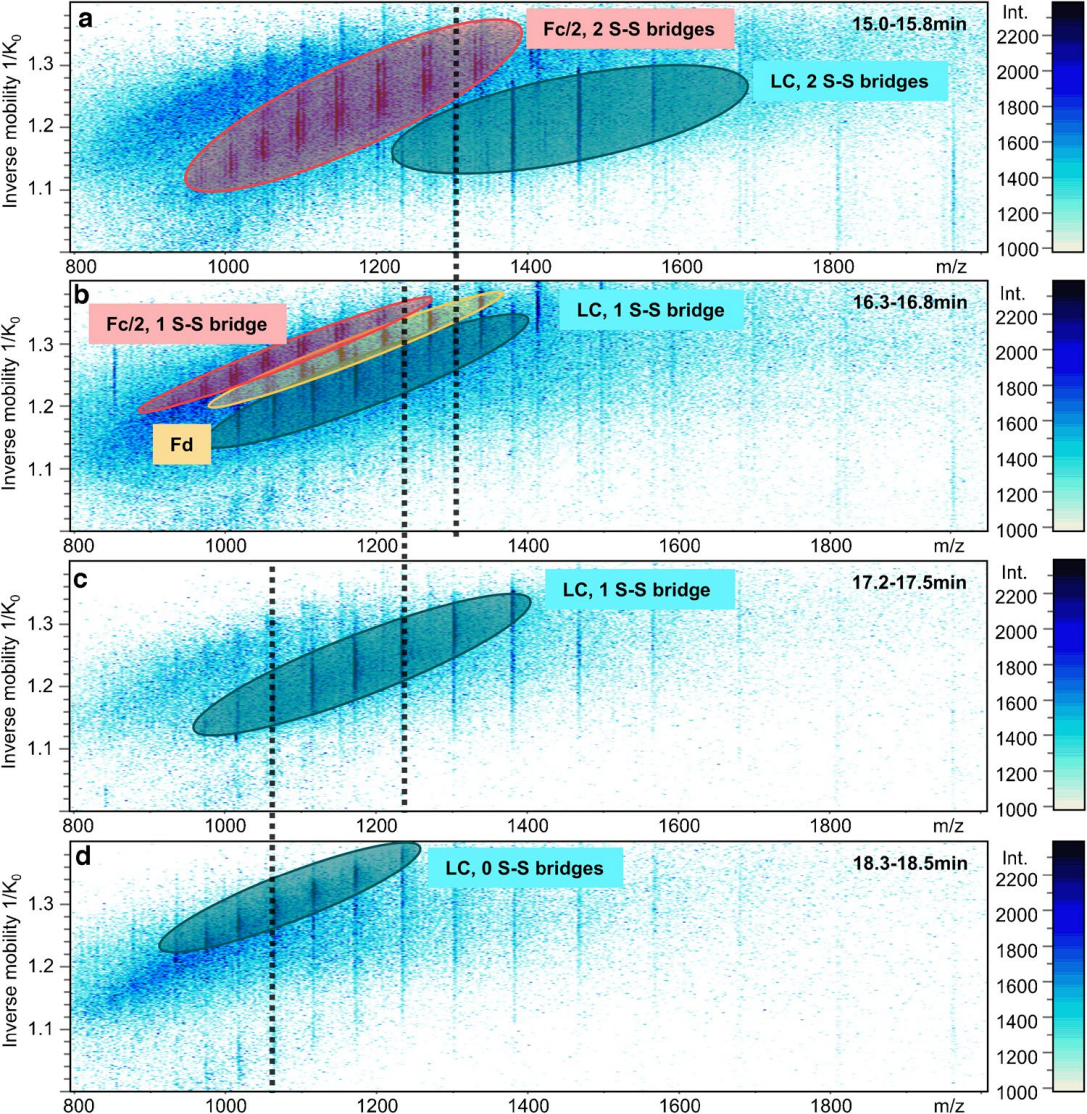
m/z-gas phase mobility heatmaps of the four migration time windows where the LC subunit of trastuzumab was detected. **a** Non-reduced LC and co-migrating non-reduced Fc/2; **b** partly-reduced LC (C134 to C194 open) with co-migrating partly-reduced Fc/2 and partly reduced Fd; **c** partly reduced LC (C23 to C88 open); **d** fully reduced LC. The black dotted lines indicate the m/z values used for the EIM generation of the various LC-subunits in Fig. 4**a**-**c**)

In another experiment, we evaluated whether the disulfides and positional isomers can be distinguished by gas phase IM-MS. The same CE system was used and coupled via the nanoCEasy interface to a timsTOF SCP. Exemplary charge envelopes plotted against the inverse mobility for the four LC forms are shown in Fig. 3.

**Fig. 4:**
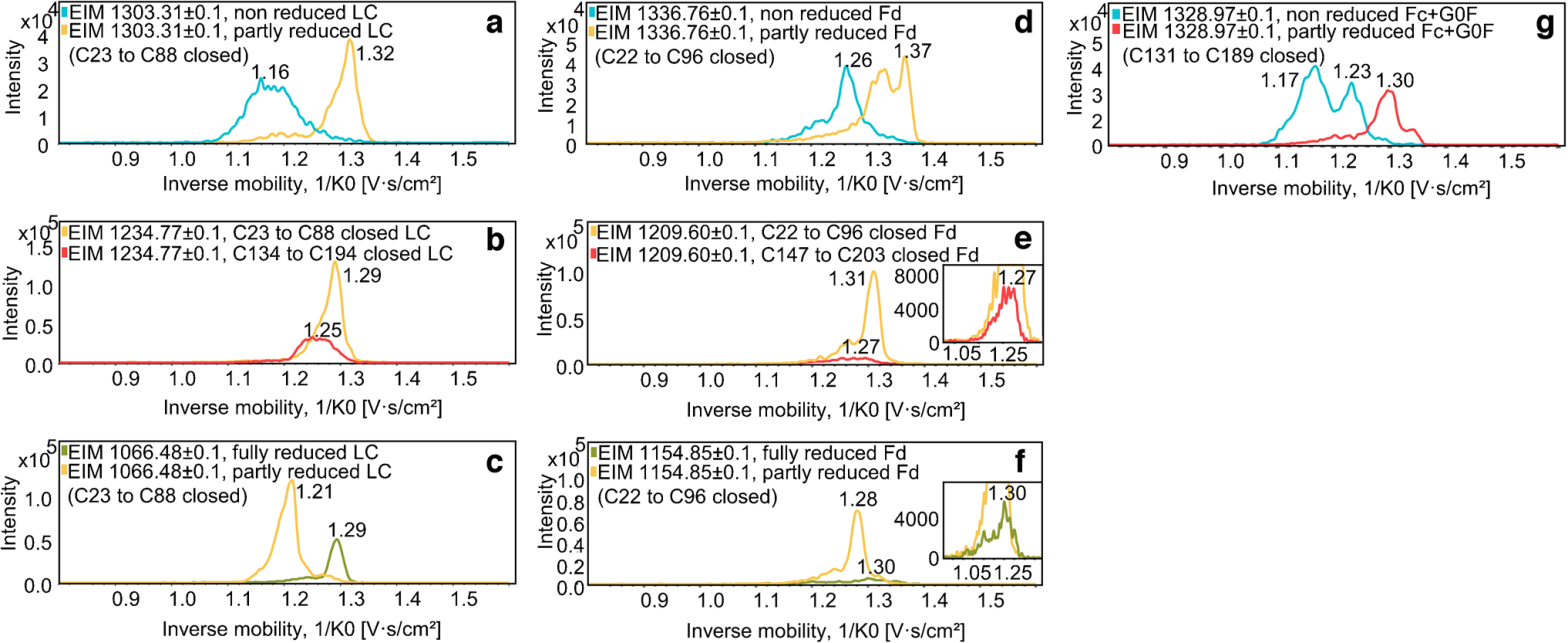
Gas phase separation of different subunits and positional isomers of LC, Fd and Fc/2. Since charge envelopes only overlap poorly, different m/z values (see Fig. 3) were chosen for comparison. Blue: non-reduced form; yellow: partly reduced form (N-terminal disulfide closed); red: partly reduced form (C-terminal disulfide closed); green: fully reduced form

The co-migrating subunit moieties described previously are visible for the different time points (Fig. 3a and b). A similar separation compared to the CE-MS/MS setup in Fig. 1 was observed, with a slight shift in migration time for all subunits. The different charge envelopes (marked by ovals) for the different reduction states are clearly visible. The non-reduced LC charge envelope (Fig. 3a) showed higher m/z values compared to the partly reduced (Fig. 3b and c) and the fully reduced (Fig. 3d) species. Within one charge envelope, higher m/z values correspond to a lower gas phase mobility. Different mobilities for the different reduction states are obtained. Non-reduced species show a higher mobility than partly and fully reduced species if the same charge state is compared. EIMs were generated for selected charge states for a more detailed analysis of the different reduction states. Since the m/z charge envelopes of non-reduced and fully reduced species barely overlap, different m/z values were chosen to generate EIMs (Fig. 4).

For all subunits, the gas phase mobility could be determined even if the charge state used for comparison was present in low intensities like one of the partly reduced Fd subunits or the fully reduced Fd subunit. For non-reduced and partly reduced LC, 1303.31 (*z* =  + 18) was chosen to generate the EIM (Fig. 4a). The non-reduced LC showed a higher mobility than the partly-reduced LC. The EIM of 1066.48 (*z* = 22) (Fig. 4c) showed that the fully reduced LC has a smaller mobility compared to the partly-reduced species. For Fd, a similar result was obtained as for the LC. The non-reduced Fd has a higher mobility than the partly reduced Fd (Fig. 4d), and even though the fully reduced species is only present in low intensities, it showed a slightly lower mobility than the partly reduced species (Fig. 4f). For Fc/2 (Fig. 4g), only fully and partly reduced species can be compared, but the same result was observed. The non-reduced Fc/2 showed a higher mobility than the partly reduced Fc/2.

The two positional isomers of LC and Fd were hardly separated in the gas phase. For the partly reduced LC (Fig. 4b), there is only a small difference in the gas phase mobility between the two isomeric species. If the disulfide bridge between C23 and C88 is closed, the molecule has a lower mobility than the LC, where the disulfide between C134 and C194 is closed. The same result was obtained for the Fd (Fig. 4e). If the disulfide bridge between C22 and C96 is closed, the molecule has a lower mobility than the Fd, where the disulfide between C147 and C203 is closed.

### Sample preparation–reduction efficiency

**Fig. 5:**
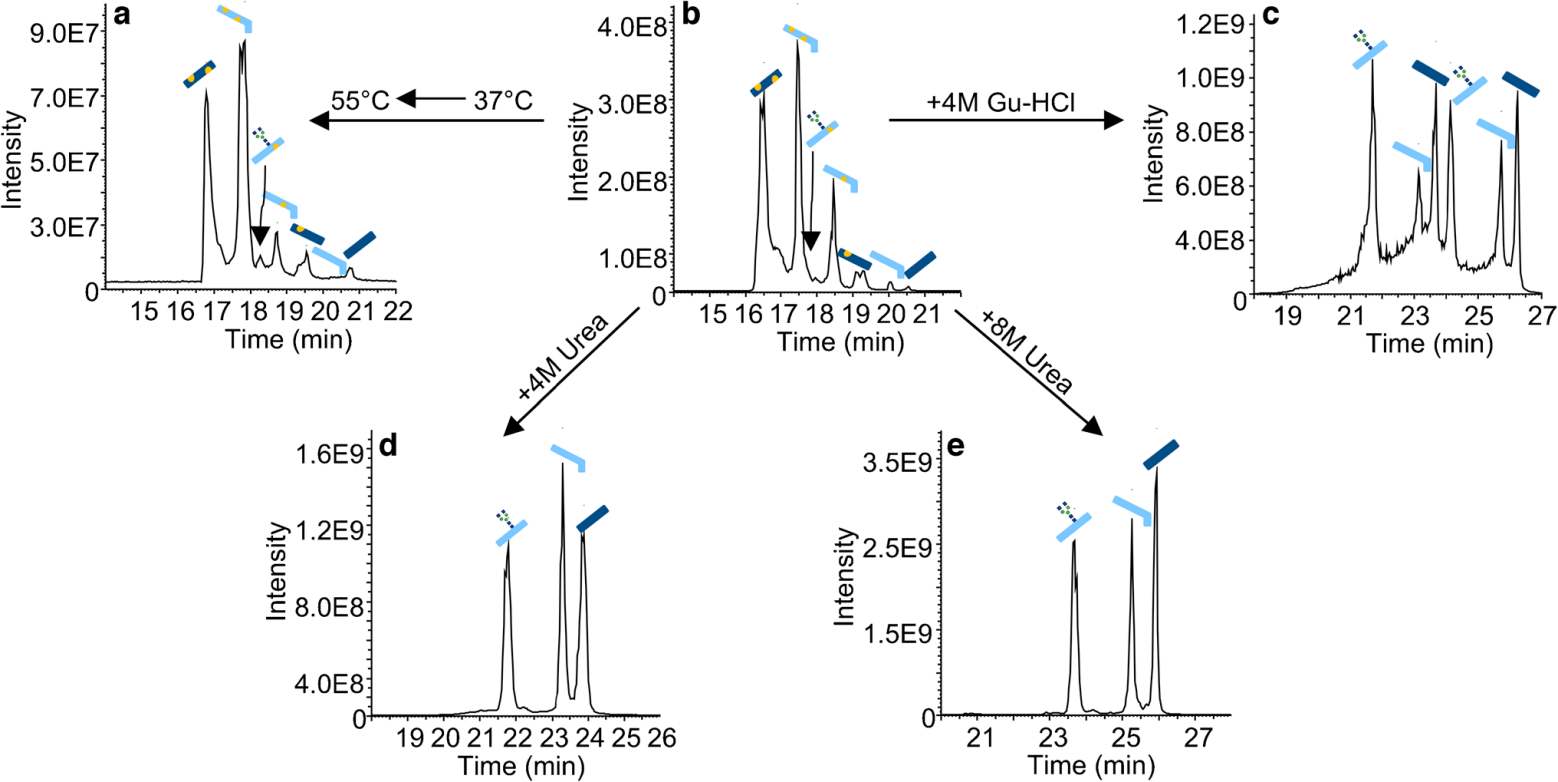
TIE of the five tested sample reduction approaches. **a** Reduction in water, 55 °C; **b** reduction in water, 37 °C; **c** reduction in 4 M GuHCl, 37 °C; **d** reduction in 4 M urea, 37 °C; **e** reduction in 8 M urea, 37 °C. The most intense subunit is illustrated with the respective symbol. Overlapping subunits are not annotated. For detailed information, see deconvoluted mass spectra in Online Resource ESM 5 (reduction in 4 M GuHCl), Online Resource ESM 3 (reduction in water 37/55 °C), and Online Resource ESM 6 (reduction in 4 M/8 M urea)

Based on the ability to characterize the reduction state of subunits in detail by applying the CE-MS/MS method developed, we tested different approaches to optimize subunit reduction. The CE-MS data of various sample preparation protocols are shown in Fig. 5. All reduction approaches were tested using the same mAb and the same enzymatic digest protocol.

In the first experiment, the reduction temperature was increased from 37 to 55 °C. As shown in Fig. 5a (see Online Resource ESM 3 for deconvoluted spectra), this did not increase the amount of fully reduced species. The TIE showed a similar peak pattern, and the signal intensity is decreased. The presence of primarily unreduced subunits was confirmed using MS/MS experiments (data not shown). Since this approach did not suffice proper reduction, a chaotropic salt was used in the reduction step, and the temperature was decreased back to 37 °C. Using 4 M GuHCl at 37 °C for 60 min, a complete reduction of all the subunit moieties (Fig. 5c) was observed and confirmed by MS/MS experiments (Online Resource ESM 4). No masses for partly reduced or non-reduced species were detected. The drawback is that the fully reduced masses for Fc/2, Fd, and LC appear at least twice in the TIE. After a Fd or LC signal, there is no return to the baseline. LC and Fd signals were present over the complete separation (Online Resource ESM 5). Therefore, another chaotropic salt (urea) was tested for complete sample reduction. Compared to the other approaches, adding 4 M urea resulted in the three expected signals in the TIE (Fig. 5d/Online Resource ESM 6). Each signal represented one subunit moiety. The subunits are fully reduced, and no partly or non-reduced species were detected even when looking specifically for these m/z values. Fragmentation coverages of 31% for Fc/2 + G0F, 34% for Fd, and 37% for LC were achieved using the EThcD fragmentation (data not shown). The migration times for the fully reduced species in the 4 M urea reduction approach are higher compared to the sample reduction in water. This correlates with an unstable but reproducible CE current that drops to 18 µA after 0.5 min and recovers to 27 µA over 15 min compared to a 27 µA stable current when no chaotropic salt was used. The migration times are even higher when 8 M urea (Fig. 5e) is used, increasing the overall analysis time. There, the current drops to 15 µA and rises back to 27 µA over 30 min. Since the sample reduction is already complete using 4 M urea, this amount of salt was used for further measurements.

### Other mAbs

After the reduction approach was optimized for trastuzumab, four mAbs (IgG1 candidates: mAb1, mAb2, mAb3, and USP mAb003) were measured with the same approach. The aim was to evaluate the general applicability of the sample preparation for other mAbs to separate and identify the states of various disulfide bridges by the presented CE-MS/MS approach (Table 1).

**Fig. 6:**
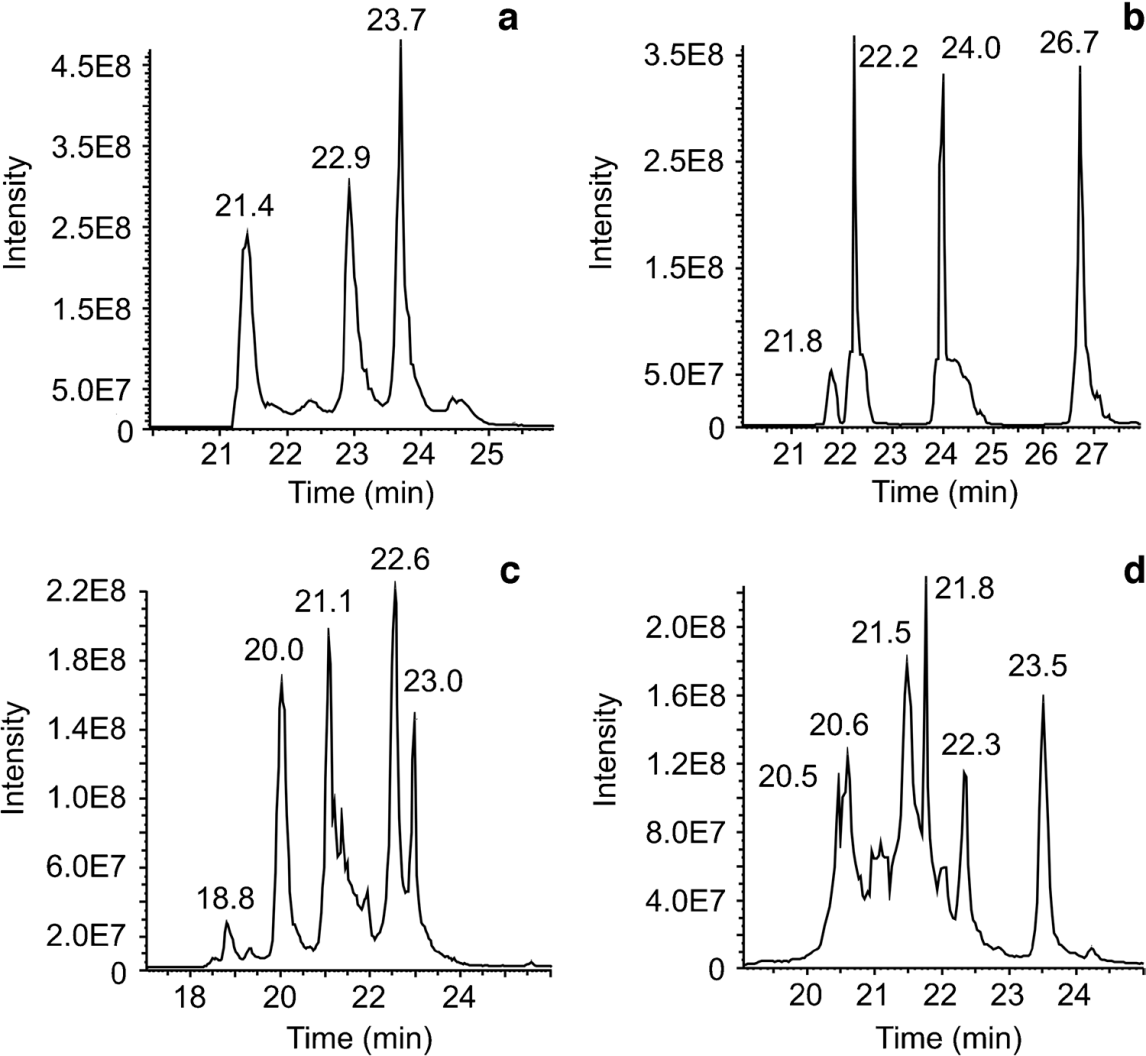
CE-MS of different mAb subunits. Identification of different disulfide bridges was done by MS/MS. **a** TIE of mAb3; **b** TIE of USP mAb003; **c** TIE of mAb1; **d** TIE of mAb2

mAb3 (Fig. 6a) and USP mAb003 (Fig. 6b) showed complete sample reduction using the optimized reduction approach containing 4 M urea. Three main signals appeared in the TIE and can be assigned by mass to the fully reduced species. Fragmentation experiments confirmed the full sample reduction. For each subunit, m/z values for fragmentation were adapted, but further fragmentation parameters were not adjusted. Sequence coverages for USP subunits of 28% (Fd) and 30% (Fc/2 + G0F, LC) and for mAb3 subunits of 14% (Fc/2 + G0F), 24% (Fd), and 30% (LC) were achieved.

mAb1 is not fully reduced using the optimized reduction approach (Fig. 6c). Small amounts of non-reduced and partly reduced species remain. The CE separates the different reduction states, and MS/MS spectra were acquired for all forms with sufficient intensities. Separating and locating the disulfide bridge for partly reduced LC and Fc/2 was possible. The partly reduced Fd was separated but identified via mass only since signal intensity was quite low. The signals of mAb1 that could be fragmented showed fragmentation coverages of 24% (Fc/2 + G0F; fully reduced), 23% (Fc/2 + G0F; partly reduced C131-C189), 35% (Fd, fully reduced), 24% (LC, fully reduced), and 32% (LC partly reduced C23-C88). Non-reduced species were not fragmented since they were only present in small amounts.

mAb2 (Fig. 6d) showed incomplete sample reduction as well. Here, the positional isomers of mAb2 showed sufficient intensities for meaningful fragmentation. The subunits that were fragmented showed fragmentation coverages of 22% (LC; partly reduced C23–C93), 13% (LC; partly reduced C139–C199), 24% (LC; fully reduced), 9% (Fc/2 + G0F; partly reduced C132–C190), 15% (Fc/2 + G0F; fully reduced), 6% (Fd; partly reduced C148–C204), 8% (Fd; partly reduced C22–C96), and 15% (Fd; fully reduced). The non-reduced species were either not present anymore (Fc/2), in low intensity (Fd), or not fragmented (LC).

## Discussion

The analysis of mAb subunits allows intramolecular disulfide characterization compared to the intact antibody analysis. We present a CE-MS system that allows the separation of the different mAb subunit moieties, as well as different reduction states of the subunit moieties (non-reduced, partly reduced, and fully reduced subunits) due to their − 4 Da, − 2 Da, and 0 Da mass shift compared to the theoretically fully reduced mass, respectively (compare Fig. 1). Sample reduction in water was executed in several publications [11, 21, 25–27, 29, 30]; however, incomplete reduction can be overseen due to insufficient separation of these proteoforms differing by only two to four Dalton. This mass shift was only seen when the reduction agent was removed prior to analysis [11, 30], when a 2D approach was applied [21], or when TCEP was used for reduction [25]. The mass shift was attributed to intact intramolecular disulfide bridges; however, no further analysis on the location of the disulfide bridge in the molecule was done. In the case of the partly-reduced subunits, our CE method can separate the disulfide bridge positional isomers. However, a CE-MS method does not determine the location of the disulfide bridge in the molecule, and there are four possibilities where the disulfide bridge could be located for each subunit. In the LC, the native disulfide bridges are between C23–C88 and C134–C194, in the Fd between C22–C96 and C147–C203, and in the Fc/2 between C25–C85 and C131–C189 (C264–C324 and C370–C428 in the HC nomenclature). If these disulfide bridges were not reduced in the first place, they should still be in their original conformation. If the bridges were reduced and oxidized again, the disulfides could be scrambled. The location of the disulfide bridges was determined for each subunit and reduction state using a CE-MS/MS approach. The positional isomers separated by CE could be identified using EThcD fragmentation with an Oribtrap mass spectrometer. In all cases where the intensities were high enough for adequate fragmentation, we could determine the exact location of the disulfide bridge in the molecule (compare Fig. 2). In these measurements, it became clear that the original disulfides were conserved and no scrambled disulfides were detected. This could have two possible explanations. First, the intramolecular disulfides of a subunit moiety show a similar tendency to be reduced [51]. If that’s the case, the light chain disulfide bridge between C23 and C88 can be reduced as easily as the disulfide bridge between C134 and C194, leading to the appearance of these two positional isomers in the sample. Second, even if both disulfide bridges are reduced, the reduced SH groups could remain in close proximity due to the lack of chaotropic salt and could rebuild their original disulfide bridge. These explanations can be applied to LC and Fd subunits, respectively. The only exception was the Fc/2 subunit, where only one positional isomer was detected. The disulfide bridge in the C_H_3-domain between C131 and C189 (C370 and C428 in HC) remained intact because it is the least susceptible disulfide to break [30, 51]. Therefore, if a disulfide bridge in the Fc/2 subunit is reduced, the C_H_2-domain bridge is more likely to be reduced, which was confirmed by the MS/MS approach. That also explains why the Fc/2 was not detected in a fully reduced form. After evaluating the different reduction states, it also became clear that non-reduced species showed poor fragmentation coverage because the intact disulfides prevented fragmentation [25]. To the best of our knowledge, the only reference that described disulfide bridge-based separation of digested and reduced mAb subunits is from Scheffler and Damoc (Application Note 72,854) using reversed-phase HPLC [25]. The different reduction states (fully, partly, non-reduced) were separated on a MabPac RP column, with subsequent identification of the positional isomers of the LC. Our approach demonstrates baseline separation of the positional isomers of all subunits and unequivocal attribution to the respective disulfide isomer using EThcD. Additionally, our approach showed a better fragmentation coverage of the subunits (Fig. 2) compared to the HCD approach of the Application Note.

In addition to the MS/MS approach, the subunits were also measured using the timsTOF SCP. CE separates molecules based on their mobility in the liquid phase. The coupling to the timsTOF adds another separation dimension. In the TIMS, the ions are transported by a carrier gas according to their collisional cross section (CCS) and an electrical field traps the molecules based on their charge. The IM-MS analysis of a peptide mix of positional disulfide isomers was previously done by Delvaux et al. [48]. While the positional isomers could be separated using CE, the drift tube IM-MS was not able to baseline separate the peptides when a mixture was analyzed. Our subunit measurements achieved a similar result. While the CE already baseline-separated the positional isomers, they were not baseline-separated in the gas phase (compare Figs. 3 and 4). However, differences in gas phase mobility can be detected depending on the reduction state. Non-reduced species showed a larger mobility than fully reduced species, which can be explained by the different shapes of the molecules due to different protein folding when disulfide bridges are oxidized or reduced. Positional isomers could be distinguished, however, requiring prior separation since gas phase mobilities differ only slightly. These experiments show the strength of the mobility separation in the liquid and gas phase. In both techniques, subunits of different reduction states can be separated. However, the positional isomers were baseline-separated using CE, while the signals for the gas phase were not baseline-separated and quite broad. Especially without separating the two positional isomers in the liquid phase, they could probably not be distinguished in the TIMS. This shows the higher resolution of the CE compared to the ion mobility separation.

For a detailed analysis of the subunit moieties, a complete reduction is beneficial to prevent sample preparation-induced heterogeneity and signal overlap, as has been the case so far. Initially, we reduced the digested mAbs in water at 37 °C for 60 min. The sample preparation in water is described in the literature a few times [11, 21, 25–27, 29]. However, a pure water-based reduction was unsuitable for complete sample reduction. The tested sample preparation approaches differed depending on the reduction conditions (compare Fig. 5). The initial increase in reduction temperature, as assumed by some studies [25, 27] does not support mAb reduction in our case. A possible explanation is that the disulfide bridges in the subunit moieties are embedded between β-sheets, hindering the DTT reduction process if no denaturing reagent is present [8, 52]. A complete trastuzumab reduction was only achieved when a chaotropic salt was used in the reduction step. GuHCl has previously been used to support the reduction of IdeS digested mAb subunits [16, 20, 22, 23, 25, 31, 33, 34]. However, in some cases, an incomplete sample reduction was still observed when samples were desalted prior to the measurement [20, 23] or the reduction was done at RT in combination with 4 M GuHCl [25]. That’s why, in our approach, the salt was left in the sample after reduction, and the reduction was carried out at 37 °C for 60 min. Even though this approach fully reduced subunits, two significant drawbacks for CE-MS may arise. First, GuHCl is positively charged in that BGE, leading to its migration towards the MS using the applied system. It causes a high background signal and contamination of the MS; however, the CE effluent can be guided to the waste due to movable CE and SL capillaries in our nanoCEasy interface [53]. In that way, the early migrated GuHCl was cut out, and the subunits reaching the MS later were analyzed without a GuHCl background signal. Second, the fully reduced subunit moieties appeared multiple times in the electropherogram. Even though it is unclear what kind of separation is observed (isomeric compounds or artifacts), GuHCl seems improper for sample reduction in the applied CE-MS system. However, that approach may work with another CE or HPLC separation system since fully reduced subunits are obtained. Another chaotropic salt is urea, which can be used for IdeS digested mAb subunit reduction [1, 24, 32]. Compared to the GuHCl approach, no salt migrates towards the MS because urea is an uncharged molecule. Therefore, the separation capillary could be positioned at the emitter tip at the beginning of the run, and no salt contaminated the MS when the nanoCEasy interface is put into conditioning mode. The light migration time shift of the urea-based approach compared to the water-based approach can be explained by the fact that urea reproducibly influences the electric conductivity of the sample zone, as observed by a drop in electric current at the beginning of the measurement. Using 4 M urea, sample reduction was complete.

Three of the five mAbs showed complete disulfide bridge reduction using the proposed sample preparation, and two mAbs showed a mixture of partly reduced and non-reduced species. One of these (mAb2) did not show the IdeS consensus sequence (CPAPELLG/GPSVF) but had two amino acids changed (CPAPELAG/APSVF). Therefore, IdeS was not cleaving the mAb at the intended location. The glycine (G) that should remain at the C-terminus of the Fd was located at the N-terminus of the Fc/2, instead. If this was considered, evaluation of subunit disulfide bridges could analogously be done to the other mAbs. The reason for the different behavior of the various IgG1 molecules could be an object to further future investigations.

In conclusion, the presented CE-MS/MS approach can be used as a fast analysis method to distinguish between complete and incomplete sample reduction. The separation and subsequent characterization/identification of the subunit variants differing in the number and position of disulfide bridges can be performed for all subunit moieties and for all tested mAbs. Ion mobility adds another separation dimension and also distinguishes different reduction states of the subunits. Complete sample reduction can be obtained using 4 M-8 M urea, whereas guanidinium hydrochloride leads to multiple signals for the same subunit of unclear origin. The here presented CE-MS approach is a valuable tool for disulfide-bridge variant characterization of mAbs on the subunit level and potentially also for other proteins.

### Supplementary Information

Below is the link to the electronic supplementary material.

Supplementary file1 (DOCX 1620 KB)

## Data Availability

The data that support the findings of this study are available from the corresponding author [CN], upon reasonable request.

## Abbreviations

BGE: Background electrolyte
CE: Capillary electrophoresis
CID: Collision-induced dissociation
C_H_1: Constant heavy chain domain 1
C_L_: Constant light chain domain
CQA: Critical quality attributes
DTT: Dithiothreitol
EIEs: Extracted ion electropherograms
EIMs: Extracted ion mobilograms
ETD: Electron transfer dissociation
EThcD: Electron transfer higher energy collisional dissociation
FA: Formic acid
FAIMS: Field asymmetric waveform ion mobility spectrometry
F(ab)_2_: Antigen-binding fragment
Fc/2: C-Terminal half of heavy chain
Fd: N-Terminal half of heavy chain
GuHCl: Guanidine hydrochloride
HC: Heavy chain
HCD: Higher energy collisional dissociation
HCl: Hydrochloric acid
HF: Hydrofluoric acid
HILIC: Hydrophilic interaction liquid chromatography
HPLC: High-performance liquid chromatography
ID: Inner diameter
IdeS: Immunoglobulin G-degrading enzyme of Streptococcus pyogenes
IgG1: Immunoglobulin G1
IM-MS: Ion mobility mass spectrometry
IPA: Isopropanol
LC: Light chain
mAbs: Monoclonal antibodies
MOPS: 3-(*N*-Morpholino)propane sulfonic acid
MS: Mass spectrometry
NaOH: Sodium hydroxide
OD: Outer diameter
PEO: Polyethylene oxide
PTM: Posttranslational modification
RP: Reversed-phase chromatography
RT: Room temperature
SL: Sheath liquid
TCEP: Tris(2-carboxyethyl)phosphine
TIE: Total ion electropherogram
TIMS: Trapped ion mobility mass spectrometry
V_H_: Variable heavy chain domain
V_L_: Variable light chain domain

## References

[1] Dadouch M, Ladner Y, Perrin C (2021). Analysis of monoclonal antibodies by capillary electrophoresis: sample preparation, separation, and detection. Separations.

[2] Kahle J, Zagst H, Wiesner R, Wätzig H (2019). Comparative charge-based separation study with various capillary electrophoresis (CE) modes and cation exchange chromatography (CEX) for the analysis of monoclonal antibodies. J Pharm Biomed Anal.

[3] Pacis E, Yu M, Autsen J, Bayer R, Li F (2011). Effects of cell culture conditions on antibody N-linked glycosylation–what affects high mannose 5 glycoform. Biotechnol Bioeng.

[4] Beck A, Wagner-Rousset E, Ayoub D, van Dorsselaer A, Sanglier-Cianférani S (2013). Characterization of therapeutic antibodies and related products. Anal Chem.

[5] Beck A, Liu H (2019). Macro- and micro-heterogeneity of natural and recombinant IgG antibodies. Antibodies (Basel).

[6] Jefferis R (2009). Glycosylation as a strategy to improve antibody-based therapeutics. Nat Rev Drug Discov.

[7] Khawli LA, Goswami S, Hutchinson R, Kwong ZW, Yang J, Wang X, Yao Z, Sreedhara A, Cano T, Tesar D, Nijem I, Allison DE, Wong PY, Kao Y-H, Quan C, Joshi A, Harris RJ, Motchnik P (2010). Charge variants in IgG1: isolation, characterization, in vitro binding properties and pharmacokinetics in rats. mAbs..

[8] Moritz B, Stracke JO. Assessment of disulfide and hinge modifications in monoclonal antibodies. Electrophoresis. 2017; 10.1002/elps.201600425. 10.1002/elps.201600425 PMC5413849 27982442

[9] Traxlmayr MW, Hasenhindl C, Hackl M, Stadlmayr G, Rybka JD, Borth N, Grillari J, Rüker F, Obinger C (2012). Construction of a stability landscape of the CH3 domain of human IgG1 by combining directed evolution with high throughput sequencing. J Mol Biol.

[10] Lacy ER, Baker M, Brigham-Burke M (2008). Free sulfhydryl measurement as an indicator of antibody stability. Anal Biochem.

[11] Giorgetti J, Beck A, Leize-Wagner E, François Y-N (2020). J Pharm Biomed Anal.

[12] Gahoual R, Beck A, François Y-N, Leize-Wagner E (2016). Independent highly sensitive characterization of asparagine deamidation and aspartic acid isomerization by sheathless CZE-ESI-MS/MS. J Mass Spectrom.

[13] Lew C, Gallegos-Perez J-L, Fonslow B, Lies M, Guttman A (2015). Rapid level-3 characterization of therapeutic antibodies by capillary electrophoresis electrospray ionization mass spectrometry. J Chromatogr Sci.

[14] Giorgetti J, Lechner A, Del Nero E, Beck A, François Y-N, Leize-Wagner E (2019). Eur J Mass Spectrom (Chichester).

[15] Zhao Y, Sun L, Knierman MD, Dovichi NJ (2016). Fast separation and analysis of reduced monoclonal antibodies with capillary zone electrophoresis coupled to mass spectrometry. Talanta.

[16] Han M, Rock BM, Pearson JT, Rock DA (2016). J Chromatogr B Analyt Technol Biomed Life Sci.

[17] von Pawel-Rammingen U, Johansson BP, Björck L (2002). IdeS, a novel streptococcal cysteine proteinase with unique specificity for immunoglobulin G. EMBO J.

[18] Dadouch M, Ladner Y, Bich C, Larroque M, Larroque C, Morel J, Bonnet P-A, Perrin C (2020). An in-line enzymatic microreactor for the middle-up analysis of monoclonal antibodies by capillary electrophoresis. Analyst.

[19] Haselberg R, de Vijlder T, Heukers R, Smit MJ, Romijn EP, Somsen GW, Domínguez-Vega E (2018). Anal Chim Acta.

[20] Gstöttner C, Nicolardi S, Haberger M, Reusch D, Wuhrer M, Domínguez-Vega E (2020). Anal Chim Acta.

[21] Stoll DR, Harmes DC, Danforth J, Wagner E, Guillarme D, Fekete S, Beck A (2015). Direct identification of rituximab main isoforms and subunit analysis by online selective comprehensive two-dimensional liquid chromatography-mass spectrometry. Anal Chem.

[22] An Y, Zhang Y, Mueller H-M, Shameem M, Chen X (2014). A new tool for monoclonal antibody analysis: application of IdeS proteolysis in IgG domain-specific characterization. mAbs..

[23] Resemann A, Jabs W, Wiechmann A, Wagner E, Colas O, Evers W, Belau E, Vorwerg L, Evans C, Beck A, Suckau D (2016). Full validation of therapeutic antibody sequences by middle-up mass measurements and middle-down protein sequencing. mAbs..

[24] Römer J, Stolz A, Kiessig S, Moritz B, Neusüß C (2021). Online top-down mass spectrometric identification of CE(SDS)-separated antibody fragments by two-dimensional capillary electrophoresis. J Pharm Biomed Anal.

[25] Scheffer K, Damoc E. Antibody subunit analysis workflow on a quadrupole-Orbitrap mass spectrometer: from optimized sample preparation to data analysis; 2018. https://assets.thermofisher.com/TFS-Assets/CMD/Application-Notes/an-72854-lc-ms-antibody-subunit-analysisan72854-en.pdf.

[26] Sun Q, Wang L, Li N, Shi L (2021). Characterization and monitoring of charge variants of a recombinant monoclonal antibody using microfluidic capillary electrophoresis-mass spectrometry. Anal Biochem.

[27] Duivelshof BL, Beck A, Guillarme D, D'Atri V (2022). Talanta.

[28] Liu T, Guo H, Zhu L, Zheng Y, Xu J, Guo Q, Zhang D, Qian W, Dai J, Guo Y, Hou S, Wang H (2016). Fast characterization of Fc-containing proteins by middle-down mass spectrometry following IdeS digestion. Chromatographia.

[29] Sokolowska I, Mo J, Dong J, Lewis MJ, Hu P (2017). Subunit mass analysis for monitoring antibody oxidation. mAbs..

[30] Belov AM, Zang L, Sebastiano R, Santos MR, Bush DR, Karger BL, Ivanov AR (2018). Electrophoresis.

[31] Ayoub D, Jabs W, Resemann A, Evers W, Evans C, Main L, Baessmann C, Wagner-Rousset E, Suckau D, Beck A (2013). mAbs..

[32] Cotham VC, Brodbelt JS (2016). Characterization of therapeutic monoclonal antibodies at the subunit-level using middle-down 193 nm ultraviolet photodissociation. Anal Chem.

[33] Fornelli L, Ayoub D, Aizikov K, Beck A, Tsybin YO (2014). Middle-down analysis of monoclonal antibodies with electron transfer dissociation orbitrap Fourier transform mass spectrometry. Anal Chem.

[34] Zhu W, Li M, Zhang J (2021). J Proteome Res.

[35] Lodge JM, Schauer KL, Brademan DR, Riley NM, Shishkova E, Westphall MS, Coon JJ (2020). Anal Chem.

[36] Shaw JB, Liu W, Vasil Ev YV, Bracken CC, Malhan N, Guthals A, Beckman JS, Voinov VG (2020). Direct determination of antibody chain pairing by top-down and middle-down mass spectrometry using electron capture dissociation and ultraviolet photodissociation. Anal Chem.

[37] Guo J, Tu H, Atouf F (2020). Measurement of macro- and micro-heterogeneity of glycosylation in biopharmaceuticals: a pharmacopeia perspective. Futur Drug Disc.

[38] Duivelshof BL, Deslignière E, Hernandez-Alba O, Ehkirch A, Toftevall H, Sjögren J, Cianferani S, Beck A, Guillarme D, D'Atri V (2020). Glycan-mediated technology for obtaining homogeneous site-specific conjugated antibody-drug conjugates: synthesis and analytical characterization by using complementary middle-up LC/HRMS analysis. Anal Chem.

[39] Bobály B, D'Atri V, Beck A, Guillarme D, Fekete S (2017). Analysis of recombinant monoclonal antibodies in hydrophilic interaction chromatography: a generic method development approach. J Pharm Biomed Anal.

[40] Verscheure L, Cerdobbel A, Sandra P, Lynen F, Sandra K (2021). Monoclonal antibody charge variant characterization by fully automated four-dimensional liquid chromatography-mass spectrometry. J Chromatogr A.

[41] Stoll DR, Harmes DC, Staples GO, Potter OG, Dammann CT, Guillarme D, Beck A (2018). Development of comprehensive online two-dimensional liquid chromatography/mass spectrometry using hydrophilic interaction and reversed-phase separations for rapid and deep profiling of therapeutic antibodies. Anal Chem.

[42] Sorensen M, Harmes DC, Stoll DR, Staples GO, Fekete S, Guillarme D, Beck A (2016). Comparison of originator and biosimilar therapeutic monoclonal antibodies using comprehensive two-dimensional liquid chromatography coupled with time-of-flight mass spectrometry. mAbs..

[43] Michelmann K, Silveira JA, Ridgeway ME, Park MA (2015). Fundamentals of trapped ion mobility spectrometry. J Am Soc Mass Spectrom.

[44] Christofi E, Barran P (2023). Ion mobility mass spectrometry (IM-MS) for structural biology: insights gained by measuring mass, charge, and collision cross section. Chem Rev.

[45] Melani RD, Srzentić K, Gerbasi VR, McGee JP, Huguet R, Fornelli L, Kelleher NL (2019). Direct measurement of light and heavy antibody chains using ion mobility and middle-down mass spectrometry. mAbs..

[46] Bagal D, Valliere-Douglass JF, Balland A, Schnier PD (2010). Resolving disulfide structural isoforms of IgG2 monoclonal antibodies by ion mobility mass spectrometry. Anal Chem.

[47] Deslignière E, Ollivier S, Ehkirch A, Martelet A, Ropartz D, Lechat N, Hernandez-Alba O, Menet J-M, Clavier S, Rogniaux H, Genet B, Cianférani S (2022). Combination of IM-based approaches to unravel the coexistence of two conformers on a therapeutic multispecific mAb. Anal Chem.

[48] Delvaux C, Massonnet P, Kune C, Haler JRN, Upert G, Mourier G, Gilles N, Quinton L, de Pauw E, Far J (2020). Combination of capillary zone electrophoresis-mass spectrometry, ion mobility-mass spectrometry, and theoretical calculations for cysteine connectivity identification in peptides bearing two intramolecular disulfide bonds. Anal Chem.

[49] Schlecht J, Stolz A, Hofmann A, Gerstung L, Neusüß C (2021). nanoCEasy: An easy, flexible, and robust nanoflow sheath liquid capillary electrophoresis-mass spectrometry interface based on 3D printed parts. Anal Chem.

[50] Iki N, Yeung ES (1996). Non-bonded poly(ethylene oxide) polymer-coated column for protein separation by capillary electrophoresis. J Chromatogr A.

[51] Liu H, Chumsae C, Gaza-Bulseco G, Hurkmans K, Radziejewski CH (2010). Ranking the susceptibility of disulfide bonds in human IgG1 antibodies by reduction, differential alkylation, and LC-MS analysis. Anal Chem.

[52] Padlan EA (1994). Anatomy of the antibody molecule. Mol Immunol.

[53] Höcker O, Knierman M, Meixner J, Neusüß C (2021). Two capillary approach for a multifunctional nanoflow sheath liquid interface for capillary electrophoresis-mass spectrometry. Electrophoresis.

