# Supplementary material for: CE-MS/MS and CE-timsTOF to separate and characterize intramolecular disulfide bridges of monoclonal antibody subunits and their application for the assessment of subunit reduction protocols
Source: Anal Bioanal Chem. 2024 Feb 1;416(7):1599–612. doi: 10.1007/s00216-024-05161-8 (PMC10899284; doi:10.1007/s00216-024-05161-8)
Supplement: Supplementary file 1 — Supplementary file1 (DOCX 1620 KB) [file 216_2024_5161_MOESM1_ESM.docx]

**CE-MS/MS and CE-timsTOF to separate and characterize intramolecular disulfide bridges of monoclonal antibody subunits and their application for the assessment of subunit reduction protocols**

Analytical and Bioanalytical Chemistry

Jasmin Schairer^a,b^, Jennifer Römer^c^, Dietmar Lang^c^, Christian Neusüß^a^*

^a^Faculty of Chemistry, Aalen University, Aalen, Germany;

^b^Faculty of Science, University of Tübingen, Tübingen, Germany;

^c^Rentschler Biopharma SE, Laupheim, Germany

Email: [christian.neuseuss@hs-aalen.de](mailto:christian.neuseuss@hs-aalen.de)


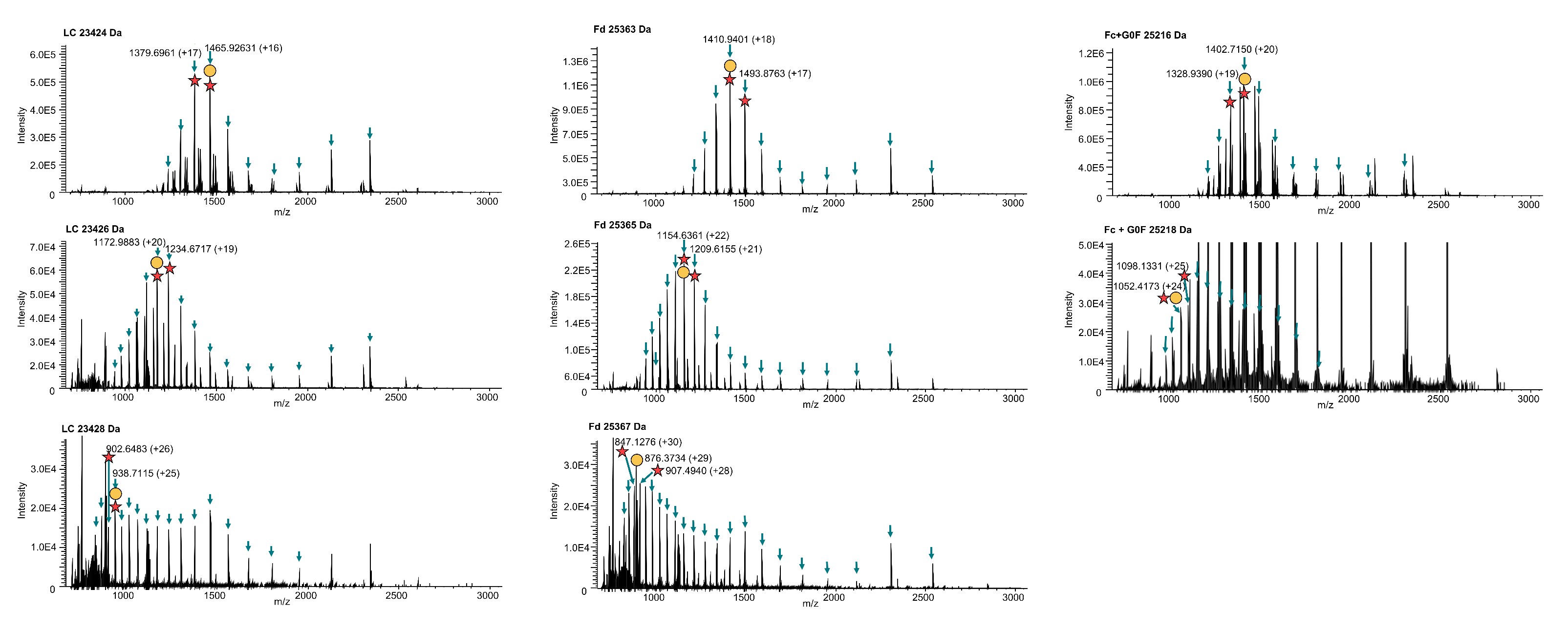


ESM 1: Mass spectra of the different subunits of trastuzumab at different migration times using the reduction in water. Blue arrows highlight the charge envelope of the respective subunit moiety. Red stars indicate the six m/z values used for summed EIE generation in Figure 1. Yellow circle shows the m/z used for EIE generation in Figure 2.

| **Subunit (closed S-S bridge)** | **CID** | **ETD** | **EThcD** | **HCD** |
| --- | --- | --- | --- | --- |
| LC (C23-C88; C134-C194) | 14% | 8% | 12% | 6% |
| LC (C23-C88) | 25% | 29% | 35% | 22% |
| LC (C134-C194) | 13% | 25% | 26% | 15% |
| LC (both open) | 20% | 21% | 21% | 21% |
| Fd (C22-C96; C147-C203) | 13% | 17% | 14% | 7% |
| Fd (C22-C96) | 20% | 24% | 24% | 18% |
| Fd (C147-C203) | 14% | 20% | 19% | 16% |
| Fd (both open) | 16% | 25% | 25% | 17% |
| Fc (C25-C85; C131-C189) | 4% | 12% | 9% | 0% |
| Fc (C131-C189) | 11% | 27% | 29% | 10% |

ESM 2: Fragmentation coverages for trastuzumab subunits after reduction in water at 37°C using different fragmetation approches. CID (35% collision energy; 10ms activation time, 0,25 activation Q), ETD (14ms reaction time; 6*10^5 reagent target; 200ms injection time), EThcD (14ms ETD reaction time; 6*10^5 ETD reagent target; 200ms ETD injection time; 12% HCD activation time) and HCD (30% collision energy) were tested.


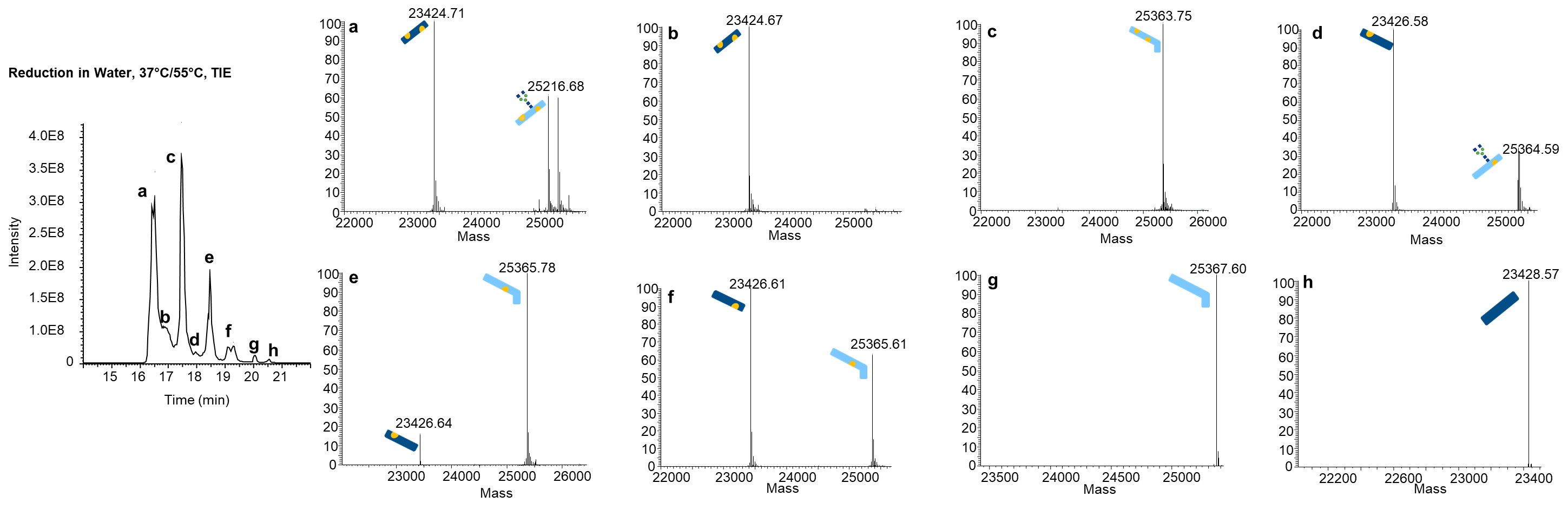


ESM 3: Deconvoluted mass spectra for reduction in water at 37°C and 55°C for each peak.


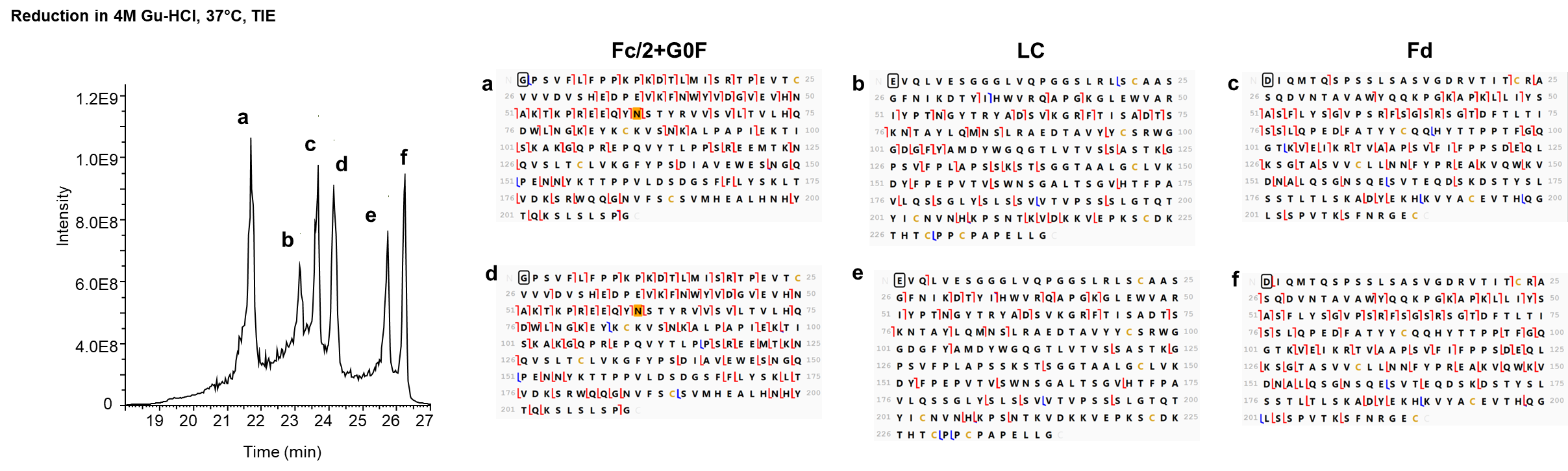


ESM 4: Fragmentation results for reduction in 4M GuHCl, 37°C for each peak


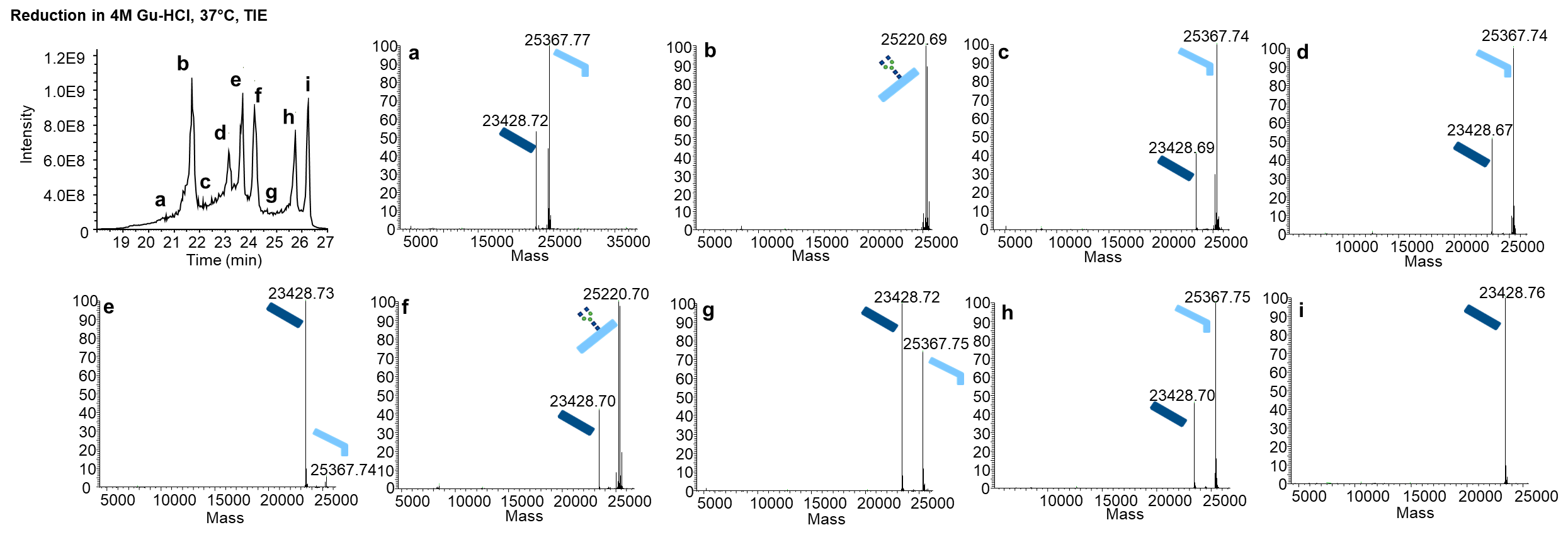


ESM 5: Deconvoluted mass spectra for reduction in 4M GuHCl, 37°C for each peak [b, d, e, f, h, i] and areas between signals [a, c, g]


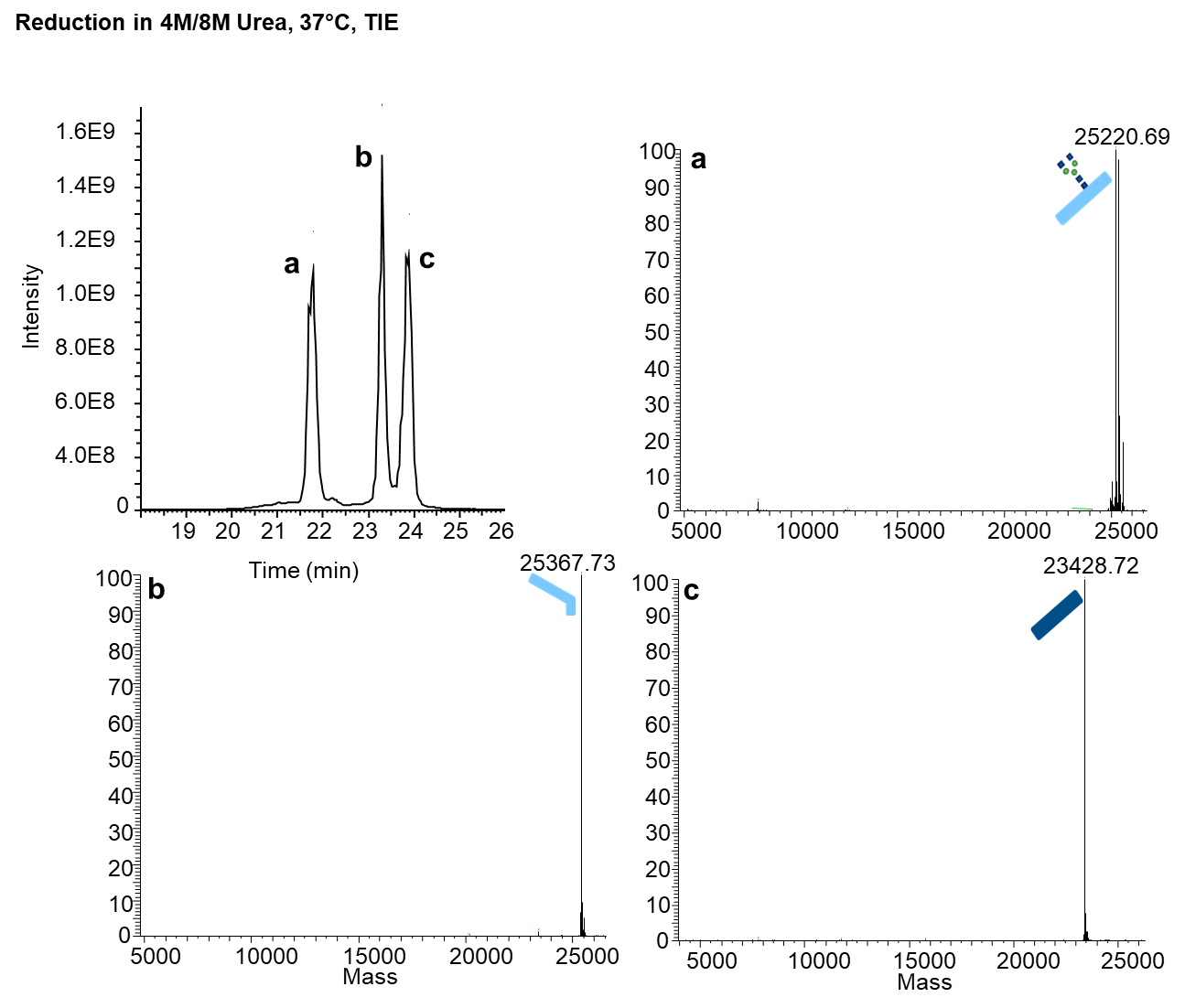


ESM 6: Deconvoluted mass spectra for reduction in 4M/8M urea, 37°C for each peak
